# Just a Few Computational Principles Generate a Realistic Model of the Brain's Visual System

**DOI:** 10.1371/journal.pbio.0040161

**Published:** 2006-04-18

**Authors:** Liza Gross

In the1950s science-fiction cult classic *Forbidden Planet*, Robby the Robot talks, cleans, learns new tasks, and understands the commands of his masters. Scientists are not at the point where they can produce Robby knockoffs with computer-driven cognition, but they are learning to use robots to probe the structure and function of the human brain. In a new study, Reto Wyss, Peter König, and Paul Verschure use a data-collecting, ambulatory robot to test a model of visual perception based on two computational functions.

When exposed to visual stimuli, neurons in the visual cortex respond to salient properties in the visual environment to create an internal representation of the world. Visual inputs are collected by the retina, sent to the thalamus, and then travel through a series of subcortical and cortical structures before reaching higher cognitive structures in the ventral visual system, including the hippocampus. In this “feed-forward” model of visual processing, neurons at different points in this visual-processing hierarchy learn to acquire increasingly complex, refined, and specific responses to these signals—even though they inhabit anatomically similar structures. There also seems to be some crossover in job duties, with evidence that the functions of specialized areas can be assumed by other regions. How the different regions of the brain acquire their specialized functions remains an open question. Is specialization an inherent trait, with each cortical region following unique computational principles? Or does each region follow the same principles and learn its specialized tasks based on its different position and input?

Theoretical neuroscientists investigate such questions by creating models to simulate the computational tasks performed by different brain structures. Such approaches have identified statistical measures called “objective functions” that can describe the computational principles of the primary visual cortex, which processes signals from the retina. For example, a statistical property that optimizes sparse representations corresponds to neurons called simple cells, while optimally stable representations correspond to complex cells. Wyss et al. asked whether objective functions could also describe the computational principles that govern the integration of visual stimuli across cortical regions.

To investigate this question, the researchers used a mobile robot programmed to navigate its environment while collecting visual inputs through a camera embedded in its circuitry. The camera provides ongoing inputs to the researcher's visual system model, which includes connections both within and between five computational units in the visual hierarchy. The model includes an unsupervised learning algorithm to optimize the stability of visual representations in feed-forward connections in conjunction with ongoing independent neuron interactions within each level—representing local memory—simulating stimulus-driven learning. The feed-forward connections also show increasing convergence, akin to that reported in the primate visual pathway.

How did the model respond to the robot-collected input? After nearly three days, all the computational levels achieved stable representations, with higher levels reaching stability only after lower levels had done so. At this point, the computational units exhibited selectivity in their response properties. Lower-level units responded to features visible from many different positions within the robot's environment and had large responsive areas that depended on the robot's orientation. Intermediate units responded to landmarks—particular views from a small region—and were highly selective for the robot's orientation. The higher units learned to link nearby landmarks, relying on small responsive regions. And the highest unit grouped these landmarks into a more complex system for representing external space—a place field—which was highly dependent on the robot's position.

The researchers used the responses of the different levels to reconstruct the position of the robot, and found that responses from the highest computational unit produced the most accurate reconstruction—in keeping with reconstructions based on the responses of rat hippocampal place cells.

These results indicate that just a few general computational principles, temporal stability and local memory, can produce specialized functions in different cortical areas. Specialization is not an intrinsic feature of these cortical areas but comes from the complex visual properties of the environment. This model of functional organization likely applies to other sensory systems, Wyss et al. conclude. If it turns out that just a few computational principles underlie higher cognitive functions as well, a real-life Robby may not be so far-fetched after all.

## 

**Figure:**
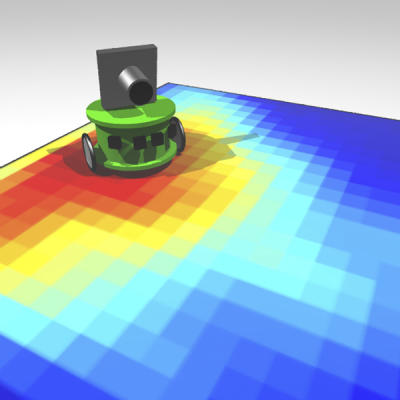
A mobile robot helped test a model of the ventral visual system based on two computational principles.

